# Local Structural Differences in Homologous Proteins: Specificities in Different SCOP Classes

**DOI:** 10.1371/journal.pone.0038805

**Published:** 2012-06-22

**Authors:** Agnel Praveen Joseph, Hélène Valadié, Narayanaswamy Srinivasan, Alexandre G. de Brevern

**Affiliations:** 1 INSERM, UMR-S 665, Dynamique des Structures et Interactions des Macromolécules Biologiques (DSIMB), Paris, France; 2 Univ Paris Diderot, Sorbonne Paris Cité, UMR 665, Paris, France; 3 Institut National de la Transfusion Sanguine (INTS), Paris, France; 4 INSERM UMR-S 726, DSIMB, Université Paris Diderot - Paris 7, Paris, France; 5 Molecular Biophysics Unit, Indian Institute of Science, Bangalore, India; King’s College, London, United Kingdom

## Abstract

The constant increase in the number of solved protein structures is of great help in understanding the basic principles behind protein folding and evolution. 3-D structural knowledge is valuable in designing and developing methods for comparison, modelling and prediction of protein structures. These approaches for structure analysis can be directly implicated in studying protein function and for drug design. The backbone of a protein structure favours certain local conformations which include α-helices, β-strands and turns. Libraries of limited number of local conformations (Structural Alphabets) were developed in the past to obtain a useful categorization of backbone conformation. Protein Block (PB) is one such Structural Alphabet that gave a reasonable structure approximation of 0.42 Å. In this study, we use PB description of local structures to analyse conformations that are preferred sites for structural variations and insertions, among group of related folds. This knowledge can be utilized in improving tools for structure comparison that work by analysing local structure similarities. Conformational differences between homologous proteins are known to occur often in the regions comprising turns and loops. Interestingly, these differences are found to have specific preferences depending upon the structural classes of proteins. Such class-specific preferences are mainly seen in the all-β class with changes involving short helical conformations and hairpin turns. A test carried out on a benchmark dataset also indicates that the use of knowledge on the class specific variations can improve the performance of a PB based structure comparison approach. The preference for the *indel* sites also seem to be confined to a few backbone conformations involving β-turns and helix C-caps. These are mainly associated with short loops joining the regular secondary structures that mediate a reversal in the chain direction. Rare β-turns of type I’ and II’ are also identified as preferred sites for insertions.

## Introduction

The three dimensional structure of protein provides precise details on its functional properties like ligand binding or catalysis [1], [2]. Protein structures can also serve as specific drug targets and structure based drug design has been quite successful. The functional properties can be studied by comparing related structures. The analysis of similarities (or variations) in protein structural features among related proteins, demands efficient means of comparing protein folds. Structural divergence occurs less rapidly than sequence divergence and structure based alignments are quite reliable when the proteins have distant relationships [3], [4], [5], [6], [7], [8], [9].

Most of the structure comparison methods consider protein folds as rigid bodies and quantify the structural similarity based on an average of atomic distances calculated using backbone coordinates. However, certain regions of a protein structure can be prone to variations, which arise due to structural flexibility or evolutionarily acquired changes. These variations can be either restricted to local regions in the backbone or involve large movements that alter the conformational state of the protein. Unlike the conformational alteration caused by large flexible movements, the local backbone changes are not likely to be affected by the nature of the global fold. Hence the preferences associated with the variations in the backbone conformations can be extracted as a general feature.

The evolutionary information has been used to explore the preferences in amino acid replacements based on empirical approaches [10], [11], [12]. Structural contexts of amino acid substitutions involving secondary structures and solvent accessibility have also been studied [13], [14], [15], [16], [17], [18], [19], [20]. Nevertheless, the precise local structural changes that occur need to be understood. Apart from local conformational changes, insertions and deletions (*indels*) seem to play a major role in protein evolution [7], [21], [22], [23], [24]. The studies on *indels* in the context of secondary structures suggested that the loops are more tolerant to *indels* than regular secondary structural regions and a significant percent of *indels* are disordered [7], [25], [26], [27], [28], [29], [30], [31]. The inserted regions prefer to be short [30] and hydrophobic amino acids were found to be less frequent in the inserted region [32]. A more detailed analysis of the effect of insertions on the flanking regions has also been carried out and insertions were found to break regular secondary structures or cause an alteration in the tertiary structure [33].

To study the preferences in the local conformational variations among homologous proteins, a good understanding of the frequent backbone conformations is necessary. The local backbone conformation of a protein chain is usually described in terms of α-helix and β-strand. More than 50% of the backbone is assigned to the coil state which reflects irregularity in the backbone. Later, more precise and comprehensive studies led to the identification of other repeating conformations [34]. The most important of them are the β-turns which cover about 25%–30% of the residues [35], [36], [37], [38], [39], [40], [41]. Out of the 9 different types of β-turns categorized based on the φ/ψ dihedrals, type I and type II are most common representing 31.6% and 10.4% of all turns (*i*.*e*., 10 and 4% of all residues). The type IV turns are comprised of those which could not be assigned to other types as per standard definitions and this has the maximum representation of about 43% [42], [43].

**Figure 1:**
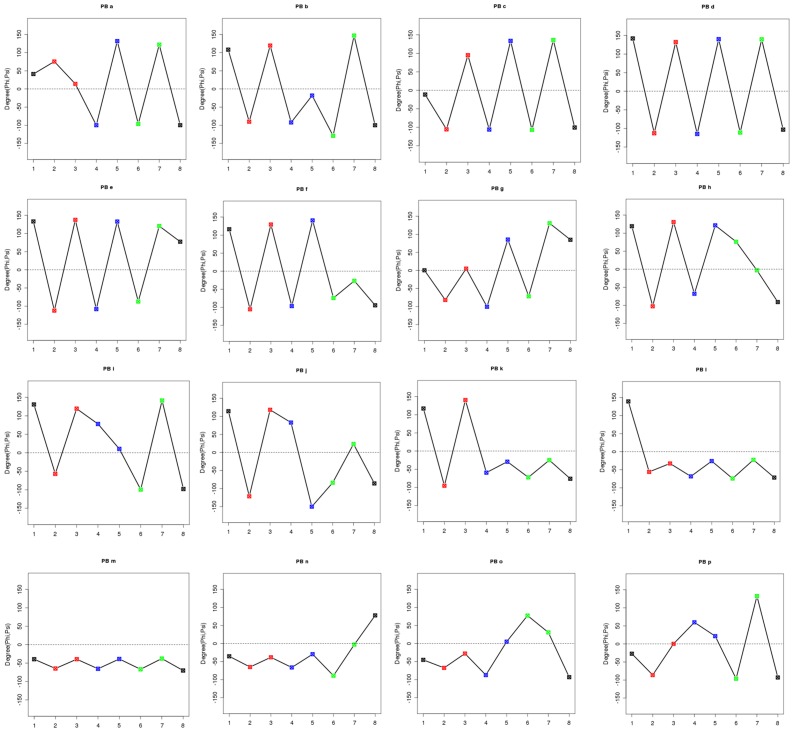
PBs series of φ,ψ backbone dihedral angles. For each PB the series of 8 dihedral angles (ψ_i−2_, φ_i−1_,ψ_i−1_, φ_i_,ψ_i_, φ_i+1_,ψ_i+1_, φ_i+2_), numbered from 1 to 8, are plotted. *i* indicates the position of an amino acid in the protein.

**Figure 2:**
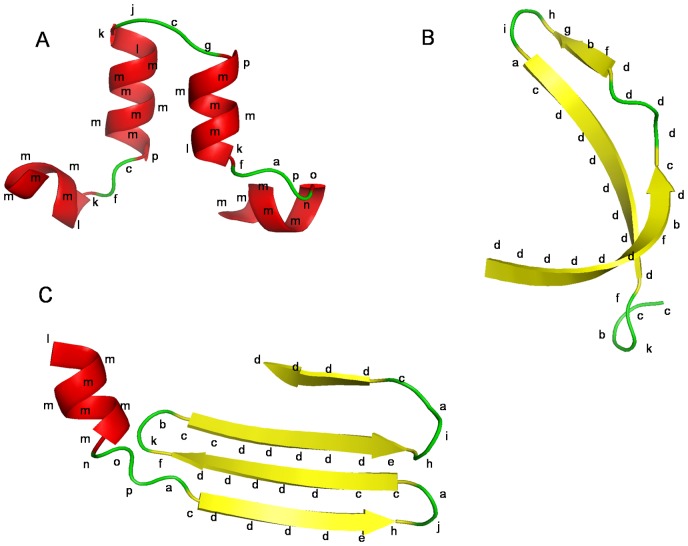
Association examples of PBs with secondary structural elements. Protein fragments (A-C) were chosen to highlight some frequently occurring PB transitions. These fragments are shown in a cartoon view distinguishing different secondary structure elements as assigned by PyMol [114]. The PB series corresponding to the local conformation of the fragment are labelled.

A more precise and different view of the favorable backbone conformations is provided by Structural Alphabets (SAs). SAs represent a library of limited number of local backbone conformations that are used to approximate the fold of a complete protein chain [44], [45], [46], [47], [48], [49], [50], [51], [52], [53]. A SA consisting of 16 prototypes called Protein Blocks (PBs) was developed in our laboratory [44], [54]. Each PB represents a pentapeptide backbone conformation described as a series of φ, ψ dihedrals and each PB is labeled by a character alphabet ranging from *a* to *p* (Figure 1). This SA gives a reasonable approximation of local protein 3D structures with a root mean square deviation (*rmsd*) of about 0.42 Å [54]. PB description has been used in several bioinformatics approaches including modeling and structure prediction [44], [55], [56], [57], [58], [59], [60], [61], [62], [63], [64], [65], [66], [67], [68], [69], [70], [71]. Figure 2 shows practical examples on the association of different PBs with regular secondary structures and Table 1 summarizes this relationship using PROMOTIF [42] based secondary structure assignment.

**Table 1:** Association of PB with secondary structures.

	H	G	E	BTI	BTII	BTIV	BTVIII	BTI’	BTII’	C	GTINV	AG	AC
**a**			25.4		14.4	17.0	2.2	1.8		29.5	1.5	4.0	
**b**			18.1	13.2		14.6	8.7		1.2	35.8	2.3		2.0
**c**		0.7	58.3	6.1		6.2	1.9			21.2	2.2		
**d**			80.4			0.8				14.4	1.2		
**e**			62.5		12.5	11.3				10.3			
**f**			38.0	11.6		10.3	3.6			31.2	2.3		
**g**	6.2	12.8	13.8	17.1	10.1	16.9	3.6			16.4	1.7		
**h**		1.6	27.2		24.4	31.7		2.1		9.8			
**i**			7.2		35.1	38.6				15.0			
**j**	8.6	2.9	10.0	3.2	3.8	22.9			9.1	32.5	1.7		
**k**	37.1	11.1		23.5	2.3	18.0				5.5			
**l**	49.1	13.0		13.5	2.3	14.0	1.9			4.3			
**m**	90.4	2.6		2.5		1.7				2.3			
**n**	66.3	6.4		6.7		10.3				7.1			
**o**	20.6	5.0		15.5	5.2	20.0		1.5		29.4			
**p**	8.3	10.8	1.4	16.7	3.1	14.7	0.8	0.9		38.5	1.2		

The percentage of different secondary structures (assigned by PROMOTIF) found associated with each PB is given. Only the secondary structures with percentage occurrence greater than 0.5% are given. The PBs are listed in the beginning of each row and the secondary structure type is given as header for each column. Abbreviation of PROMOTIF assignments: BT*X* – β-turns, X is the type of β-turn, AG – Antiparallel strands, G1 type β-bulge, where the first residue is in the left handed helical conformation (usually Glycine), AC – Antiparallel strands, Classic type beta bulge, one extra residue forms the bulge, GTINV – Inverse γ-turns (φ = −79.0±40,ψ = 69.0±40).

As in the case of the study of amino acid substitutions that occur during the course of evolution, the preferred local structural changes could be analysed with the help of PBs. This idea was extended to the comparison of protein structures. Approximation of protein structures in terms of SA helps to transform 3D information in 1D. Thus the 3D superposition of protein structures can be carried out with an alignment of sequences encoded in terms of SAs [67], [72]. A specialized PB substitution matrix (SM) was developed for this purpose [73]. The PB based structure alignment approach performed better than many of the other available tools for structure comparison [67], [74].

In this study we analyse the preferences for the conservation of local backbone conformations with the help of Protein Block abstraction. Initially, we analyse the pattern of PB substitutions and the effect of solvent accessibility on this. Here, we restrict our analysis to the equivalent structural regions found among families of related folds. This knowledge can be utilized in the improvement of structure comparison tools that works based on the similarities in the local backbone or fragment conformations. As the secondary structure content and topology varies between structural classes of proteins (as defined by SCOP [75]), we check whether there are class-specific specificities for changes in local pentapeptide conformations. In that case we also verify the use of class specific PB substitution matrices in improving the alignment of structures represented in terms of PB sequences. The preferred local backbone conformations associated with the sites of insertions were studied. Throughout the study, we associate the PB description of backbone conformation with different secondary structure assignments, to present a different view of the results.

## Methods

### Protein Blocks

Protein Blocks (PBs) are a set of 16 prototypes of main chain conformations that are 5 residues long. The pentapeptide backbone conformation is described in terms of the φ, ψ dihedral angles. The 16 prototypes are labeled from *a* to *p* (Figure 1). They were generated using an unsupervised classifier related to Kohonen Maps [76] and hidden Markov model. Protein Blocks renders a reasonable approximation of local structures in proteins [44] with an average root mean square deviation (*rmsd*) of 0.42 Å [54]. The assignment of PBs [54] has been carried out using an in-house Python software similar to the one used in iPBA web server [77].

Figure 2 highlights the correspondence between PBs and regular secondary structures assigned by DSSP (Dictionary of Secondary Structure of Proteins) [43]. The PBs *m* and *d* are prototypes for the central region of α-helix and β-strand, respectively. PBs *a* through *c* primarily represent the N-cap of β-strand while *e* and *f* correspond to C-caps. These N and C caps could also include regions in the loop leading to or arising from a secondary structural element. The PBs *p*, *a*, *f*, *h, g* and *i* are often seen in the region of transition between secondary structural elements. Figure 2A–C presents some examples highlighting the association of the PB structures with respect to the secondary structure definition while Table 1 gives a detailed list of this relationships extracted from a subset of PALI (Phylogeny and ALIgnment of homologous protein structures) [78] dataset generated using a sequence identity cut-off of 40%. Figure 2 also highlights some of the frequently occurring PB-PB transitions. PBs *g* through *j* are largely associated with coils, PBs *k* and *l* are frequent in the N cap of α-helix and *n* to *p* in C-caps.

### Dataset

The dataset of protein structure alignments used in the study is the recent version of PALI dataset V 2.8a [78], [79], [80]. It consists of 1,922 domain families comprising of 231,000 domain pairs aligned using MUSTANG [81]. The domains are classified based on SCOP definitions [75]. SCOP classifies domain structures into four major classes. All-α class consists of proteins with mainly α-helical content while all-β proteins are composed of mainly strand conformation. α/β contains both helical and strand conformations that are mixed in the structure, while they are segregated in the case of α+β class.

### PB Substitution Matrix

Domain pairs in the PALI database that are solved at resolution better than 2 Å and share sequence identity less than 40%, were only used for obtaining the substitution frequencies. This corresponds to 5,223 domain alignment pairs from 476 families. The pairwise structural alignments were first represented as PB sequence alignments. The PB pairs occurring in the structurally conserved regions (within 3 Å) were counted for calculating the substitution frequencies. As in our previous work [72], the method presented by Johnson *et al*. [82] was adopted for calculating log odd scores from raw frequencies:
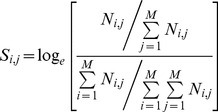
(1)where *S_i,j_* is the substitution weight and *N_i,j_* is the raw substitution frequency between PB *i* and PB *j*, *M* is the total number of different PBs (*i*.*e*., 16).

### Structural Superposition Based on PBs

Protein structures to be aligned were first represented as PB sequences. These sequences have been aligned using Smith-Waterman dynamic programming algorithm [83], based on the PB substitution scores. Gap penalty of −5.0 was used for alignment [67]. Profit version 3.1 [84] was used to obtain a least squares fit of two protein structures based on the PB sequence alignment. The amino acid sequence alignment corresponding to the PB alignment was given as input for Profit for reading the aligned pairs of residues. The fit was performed on the aligned residue pairs and the Root Mean Square deviation (*rmsd*) was calculated.

### Test Dataset for Alignments

The gain in the quality of superposition (quantified as the difference in *rmsd* of superimposition) obtained using the class specific PB substitution matrices was checked on a smaller dataset. From each SCOP superfamily in the PALI dataset (with two or more families), two families were randomly chosen and from each of these families, a domain pair with sequence identity less than 40%, was chosen. It represents 1,050 domains (comprising of 188,760 residues) from 263 families.

### Clustering Based on Substitution Data

To compare the PB substitution patterns, pairwise correlation coefficients were calculated based on the substitution scores associated with each PB. These values were deducted from 1 to get a distance matrix for hierarchical clustering. The *hclust* module of ‘R’ software (http://www.r-project.org/) was used for clustering the PBs based on the distance matrix.

### Secondary Structure Assignment

The secondary structure types associated with the PBs were identified with the help of assignments made by DSSP [43], SEGNO [85] and PROMOTIF [42].

### PB Accessibility

A PB is considered solvent accessible if at least 3 residues (out of 5) that it corresponds to, are accessible to the solvent. NACCESS [86] was used for calculating the accessibility of each residue. Different cut-offs of 7%, 15% and 25% for relative solvent accessibility, were used to identify buried residues.

### Locating Indels

The structural alignments of domain pairs sharing less than 80% sequence identity cut-off were extracted from PALI. If a continuous stretch of gaps of length *n* is flanked by aligned regions (each aligned residue pair within 3 Å) that are at least 3 residues long, then that position is considered as a point of insertion/deletion.

### Z Value

A likelihood score was computed to identify significant members of a distribution. This was used to identify the local conformation prone to insertions. The preferred series of two PBs (di-PBs) binding the insert site are extracted from the observed distribution of di-PBs. The background frequency of occurrence of di-PBs in the dataset was considered as the expected distribution. Z values were computed based on the deviation from the expected distribution. The di-PBs with Z values greater than 2 were considered as the preferred sites for insertions.

## Results

The extent of conservation of local backbone conformations were identified in terms of PBs. The local structures undergoing subtle conformational differences and those which are preferred as insert sites, were looked into. Pairwise structural alignments from the PALI dataset were used as a reference to study such preferences among related structures in a family.

### Local Structure Substitutions

The changes in local backbone conformation were deduced by looking at PB replacements among homologous structures. The reliable alignment regions (residue pairs within 3 Å) are only considered for calculating the replacement frequencies. The scores for substituting each PB with the 16 PBs, were calculated from the raw substitution frequencies (see *Methods*).

**Figure 3:**
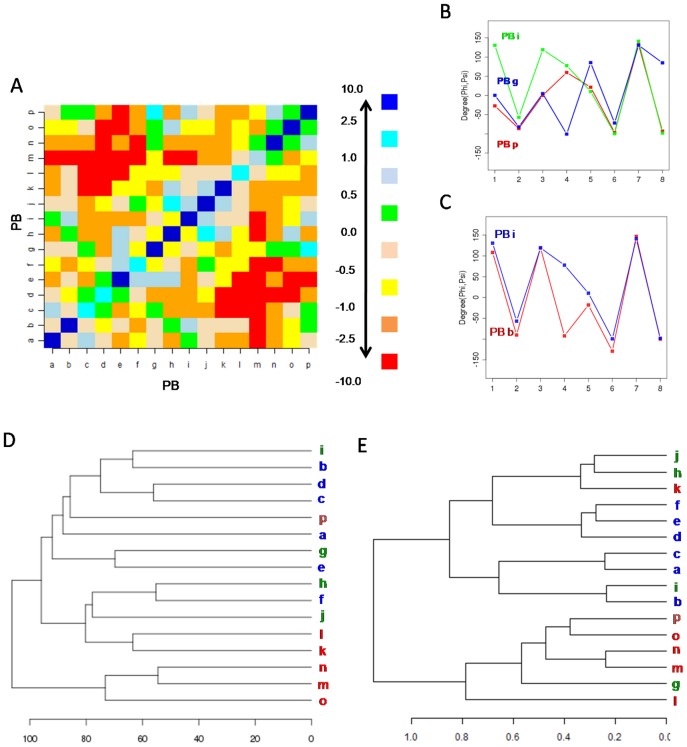
PB substitutions. (A) The variation in substitution score in the PB substitution matrix is highlighted using a colour-code, as shown. (B) The series of dihedral angles (ψ_i−2_, φ_i−1_,ψ_i−1_, φ_i_,ψ_i_, φ_i+1_,ψ_i+1_, φ_i+2_), associated with the PB substitutions (*p*, *g)* and (*p*,*i)* and (C) (*b,i*). These represent some of the preferred local conformational changes (D) Hierarchical clustering of PBs based on the similarity of dihedral angles, measured in terms of angular *rmsd*. The PBs frequently associated with helices are in red, those found often with beta strands are in blue and the rest are in green (E) Clustering of PBs based on the substitution pattern associated with each PB (see *Methods*).

Figure 3A shows the substitution preferences associated with each PB. Surprisingly, the PBs associated with the N and C caps of helix and strand do not show highly preferred substitutions with the central helix PB *m* and central strand PB *d* respectively. This reflects the preference for conservation of the central or most favoured conformation of these regular structural elements. The PB *p*, usually found in the C-cap of helices and/or at the N-cap of β-strands, favours substitutions with PBs *g* and *i*. The PB pairs (*p*, *g*) and *(p, i*) share similar (φ,ψ) dihedrals along the 5 residue stretch (see Figure 3B which compares the dihedral angles associated with these PBs). The substitution (*p*, *g*) is dominated by changes in conformation of 3._10_ helices and β-turns and a relatively fewer conversions to α-helix and coil (Table 1, Figure S1 & Table S1). These turns are mainly characterized by β-turns of type I and IV. On the other hand, (*p,i*) substitution involves variations in turns (β-turns type I, II and IV) and the substitutions between them and coils. These two substitutions mainly involve the region of helix-helix, strand-strand and helix-strand transitions (Figure S1). PB *b* which is largely seen in the N cap of β-strands, favour replacement with PB *i* which is frequently seen in the region of strand-strand transitions (Figure 3C). This change is associated with variation in turns and bends, mainly involving transitions between β turns of types I, & IV with types II and IV.

It is expected that the preference for PB substitution is dependent on the extent of structural similarity between PBs. Nonetheless, often the structurally closest PBs are not the ones with the best substitution preference (Figures 3D&E). For instance, the substitution of PB *f* and PB *h* is not high preferred (Figure 3E), even though they are very close in terms of the dihedral angle distribution. The preference for replacement can be dependent on the local structural environment. This is also true in the case of substitutions (*k*, *l*) and (*c*,*d*), which are not highly favoured even though they are structurally closest. PB *j*, which is usually seen in coils, favours replacement with *h* (Figures 3A and S2). PB *k* associated with N-cap of helices, also show preferred substitution with the loop PB *h*. These two changes are characterized by variations in β-turns and 3._10_ helices (Figure S1). The replacement of *h* and *i* which are largely seen in the strand-strand transitions, with central α-helix PB *m* is strongly disfavoured. The more obvious case involving substitutions between helix and strand associated PBs, are not preferred (Figure 3A).

Hence many of the preferred variations in the backbone conformation, corresponds to changes in β-turns. The clustering based on the substitution pattern of each PB (Figure 3E) highlights differences with respect to the association based on PB conformation similarity (Figure 3D). The PBs associated with the helical conformation, *i.e. l* (N-terminus), *m* (central) and *n, o* and *p* (C-terminus) have similar preferences for substitution. PB *k* which is also frequent in the N-cap of helices has patterns of substitution similar to the loop associated PBs (*j,h*). On the other hand, the PBs mainly occurring at the N-terminus of strands cluster separately from the rest of strand associated PBs.

It should be noted that there are significant variations in the substitution preferences, among the helix associated PBs and those associated with the strands. The PBs associated with the central region of helix and its immediate C-terminus, *i*.*e*., PBs *m* and *n* are found to group closely. Similar relationship is observed in case of strand associated PBs *d*, *e* and *f.*

As mentioned in the *Methods* section, the local conformational changes discussed above were identified using a dataset of domain pairs sharing less than 40% sequence identity. To check whether the nature of backbone conformational changes has significant differences depending on the extent of structure relatedness, we compared the substitution patterns obtained from datasets filtered at different sequence identity cut-offs like 60%, 80% and finally a dataset with all domain pairs (no filtering, Figure S3). No significant differences were observed with respect to the original dataset (filtered at 40% sequence identity), the PB substitutions had correlation scores close to 1.

### PB Substitution and Accessibility

**Figure 4:**
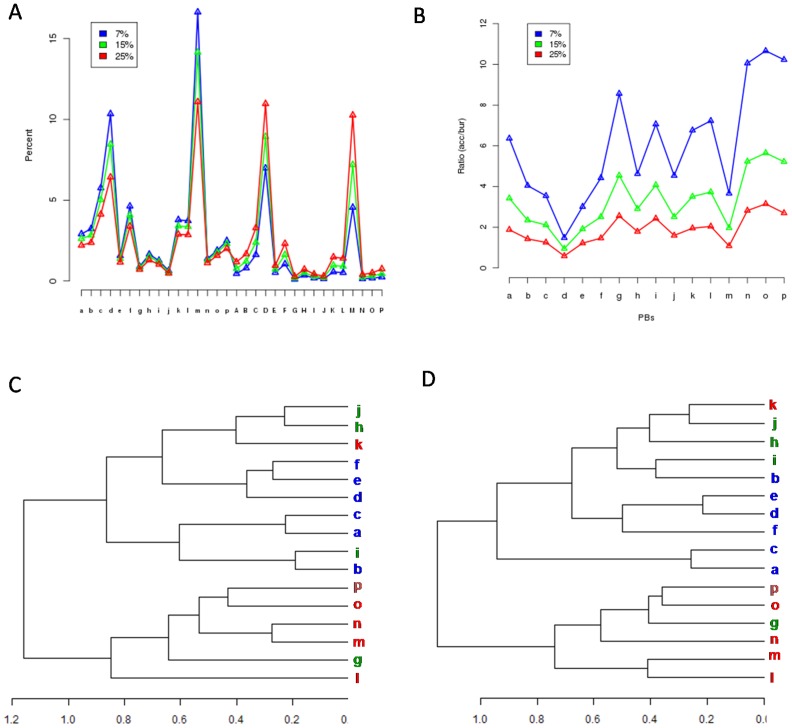
Clustering PBs based on substitution patterns. (A) Distribution of accessible and buried PBs classified based on different accessibility cut-offs of 7%,15% and 25%. Ratio of frequency of exposed PBs to that of buried, plotted for each of the 16 PBs (B) Hierarchical clustering of PBs classified as exposed (B) and buried (C) at an accessibility cut-off of 15%. The clustering is based on the correlation of substitution scores.

Each PB was first classified into accessible and buried (*see Methods*) and the occurrence frequency was calculated. Figure 4A gives the ratio of the percentage of accessible PBs to buried. PB *d* found at the central strand regions, has the highest tendency to get buried (Figures 4A&B). The helix associated PBs has a higher preference for solvent exposure than that of the strand associated PBs. The PBs associated with the C-terminus of helices (*n*, *o* and *p*), have a greater tendency to get exposed when compared to the N-cap. On the other hand, both the N and C caps of strands have similar preferences for exposure. The loop associated PBs has variable preferences, with *g* and *i* being more accessible than *h* and *j.* The PB *g* is dominated by short helical conformations (including 3._10_ helices) and turns, while PB *i* is very frequent in turns (Table 1). The relative increase in exposure with increase in the threshold for burial also shows a similar trend. The strand associated PBs have a relatively lower increase in the percentage of exposure.

It is interesting to find out whether the substitution patterns vary with solvent accessibility of the local structures. To apprehend it, a substitution matrix was generated for the PBs categorized as exposed and buried (Figure S4). Apart from a few exceptions, the distribution of scores for substitutions between exposed PBs and between buried PBs was largely similar to the general distribution (Figure 3A). Substitution (*k*, *i*) is preferred in the buried regions than exposed. Most of the substitutions involving the replacement of an exposed PB by a buried PB of another kind are not favoured. The substitutions (*p*, *g*) and (*h*, *j*) are exceptions.

Clustering exposed and buried PBs based on the substitution patterns suggests that PBs associate differently depending on their accessibility (Figures 4C and D). The exposed PB (Figure 4C) cluster in a way similar to the general preferences (Figure 3A). In the buried region, the PBs *b* and *i* cluster with the loop PBs and not with the strand associated PBs. The substitution patterns associated with the central helix conformation *m* is not highly similar to the substitutions in the immediate C-terminus (PB *n*), unlike the exposed regions.

### Class Specific PB Substitutions

The distribution of domain structures in different SCOP classes is based on the secondary structure content and topology. As a result, the background distribution of PBs also varies between the SCOP classes. For instance, the all-α class has very low percentage of strand associated PBs while all-β has a low percentage of helix associated PBs (Figure S5).

The PB substitution scores observed in the different SCOP classes were compared to the scores observed in the global distribution. The PB substitution patterns show variations across different SCOP classes. Clustering PBs based on the substitution patterns reflect different behaviours in each structural class.

**Figure 5:**
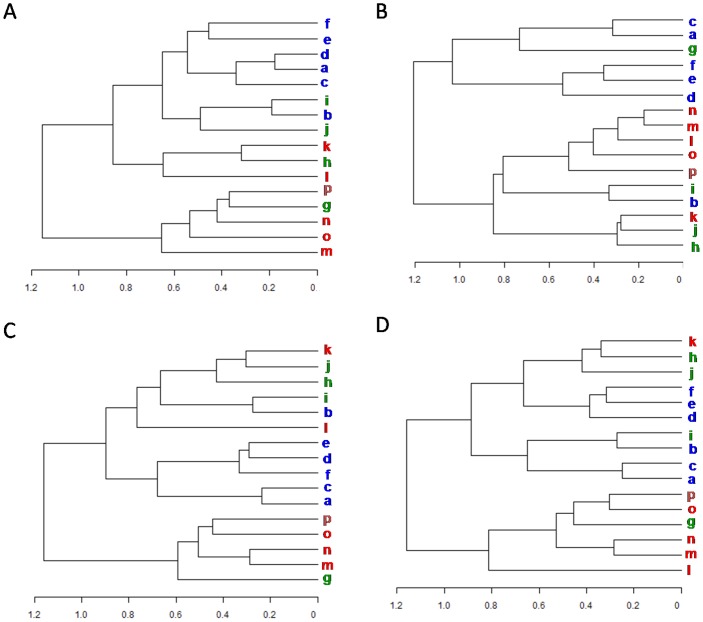
PB relationship in each SCOP class derived based on the substitution pattern. (A–D) Hierarchical clustering of PBs based on substitution patterns specific for each SCOP class. The clusters correspond to relationships observed in all-α (A), all-β (B), α/β (C) and α+β (D) classes.

For the all-α class (Figure 5A), the PBs mainly occurring in helix N-terminus, is associated with loop PB *h* which is largely found in β turns and strand C terminus. For the all-β class (Figure 5B), the group of loop associated PBs cluster is closer to the helix PBs than those which correspond to the strand.

The PBs in the α/β class (Figure 5C) associate in a similar fashion as that of the global distribution, except that the PBs *a* and *c* which mark the beginning of strands, cluster closely with the other strand PBs and the helix N cap PB *l* associates with loop PBs. The clustering in the α+β class (Figure 5D) is closest to the general distribution (Figure 3D).

#### Preferred substitutions in each class

**Figure 6:**
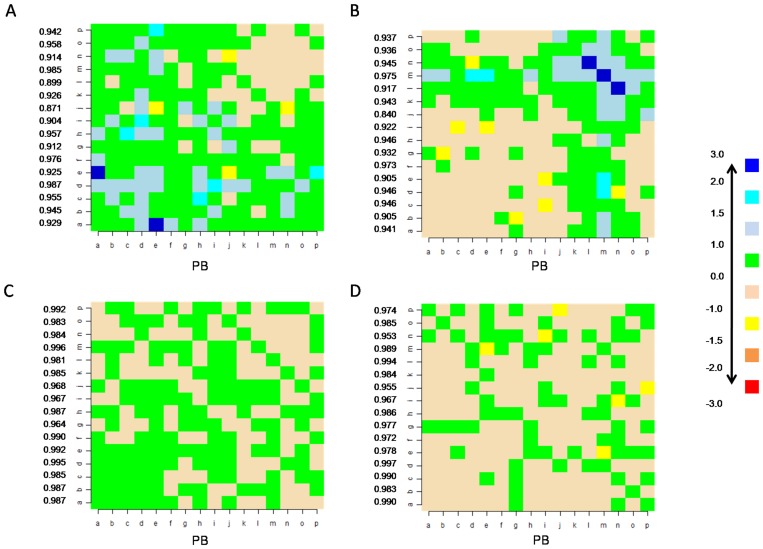
Comparison of class-specific PB substitution scores with the global distribution (global substitution matrix). The differences in the PB substitution scores specific for the all-α (A), all-β (B), α/β (C) and α+β (D) classes, with respect to the global matrix, are plotted. The correlation coefficients obtained by performing row-wise comparisons (class-specific PB substitution patterns *vs* Global) are also indicated adjacent to the difference matrices.

Thus variations in the substitution preferences of local structure conformations are seen across SCOP classes. Comparison of these class-specific substitution scores with the global matrix (*see Methods*) highlights a few differences (Figure 6).

It was seen that substitutions involving strand associated PBs and helix associated PBs have a higher score in the all-α and all-β classes respectively (Figures 6A and 6B). Indeed, they have lower background frequencies or lack sufficient substitution information in these respective classes. Nevertheless, the observed probabilities of changes between strands associated PBs with the central conformation *d* was low in the all-α class. Similarly, in the all-β class, the substitutions involving central helix conformation *m* and other helix associated PBs have low probabilities of occurrence (Figure S6). More class specific preferences for the change in local conformations were evident in the all-α and all-β classes (Figure 6). The substitution patterns associated with each PB was compared with that of the general preferences (Figure 3A) and the cases where the correlation was less than 0.95 were looked into.

**Figure 7:**
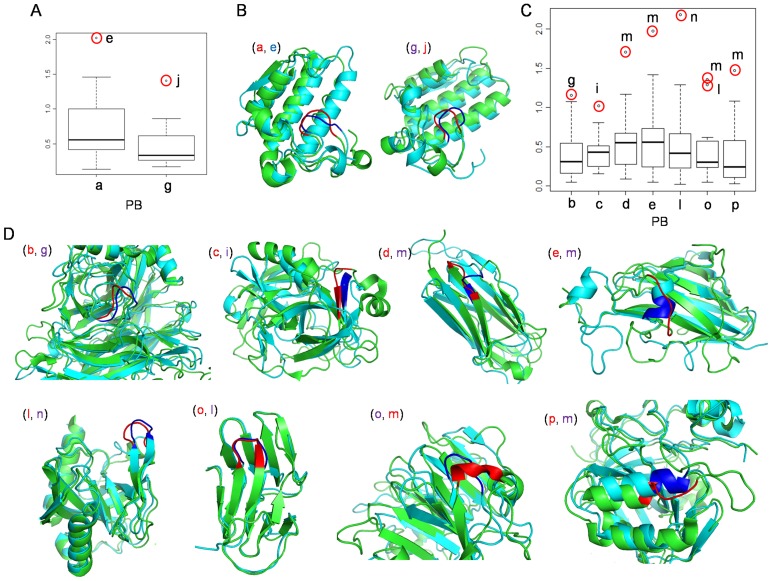
PB substitutions highly preferred in certain SCOP classes. The cases where the class-specific substitution scores associated with each PB (each row in the substitution matrix) has a correlation less than 0.95 when compared to the global matrix, were looked into. The absolute differences (class specific *vs* Global) of substitution patterns (respective rows) were plotted as a boxplot, to identify outliers. Substitution scores lying outside a 1.5 inter-quartile range (IQR), were considered as outliers or significantly different from the global substitutions. For the all-α class, (A) the plots are generated for PBs *a* and *g*. (B) highlights examples of backbone conformations corresponding to substitutions detected as outliers. Similarly, boxplots were generated for the all-β class (C) and the examples of significantly different substitutions are shown (D).

In the all-α class, two substitutions (*a*, *e*) and (*g*, *j*) were found to be more favourable when compared to the global preferences (Figures 7A&B). Both the substitutions are usually associated with changes in β-turn type II, II’ and type IV conformations.

The substitutions that are preferred in the all-β class occur in the region of strand-strand transitions (Figures 7C&D). These substitutions can be grouped into the following categories. (i) Those which involve transition between central helix conformation (PB *m*) and those frequently associated with strands (PBs *d* and *e*). This change is usually characterized by changes in short helical regions found in this class. (ii) Those usually associated with beta turns. This includes PB changes (*b,g*), (*c,i*), (*l,n*) and (*o,l*) in the regions which are mainly characterized by hairpin beta turns.. (iii) Those associated with transitions between central helix and C-terminal PBs. The substitutions (*o*,*m*) and (*p*,*m*) belong to this category.

### Sites of *Indels*

**Figure 8:**
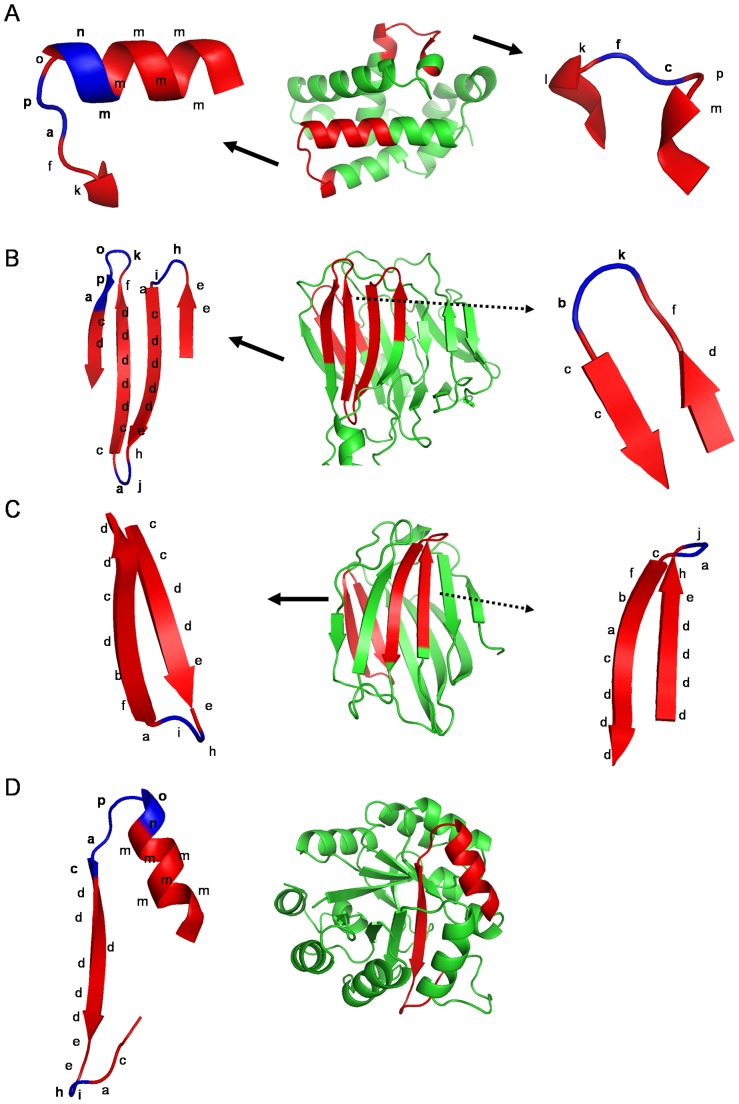
Preferred local structure for indel events. The di-PBs that bind the site of insertions are shown in the context of secondary structure definition. Parts of four domain structures (A–D) are used to highlight the indel sites.

The sites of insertion/deletion events were analysed using PBs. The frequencies of the two PBs (di-PBs) that bind the site of *indels*, were calculated (*see Methods*). Preferred sites of insertions were identified using Z-values. The local structural regions where *indels* occur show some preferences (Table 2 & Figure 8). The length of the insert also affects the preferences for the insert site. However, certain di-PBs like ‘*p-a*’ and ‘*j-a*’ are the preferred sites for insertions of different lengths.

**Table 2:** Preferred *indel* sites in different SCOP classes.

SCOPClass	InsertLength	Insert sitePBs(i,i+1)	PB series	Promotifassignment	φ_i_,ψ_i_; φ_i+1_,ψ_i+1_
**All-α**	1	MN	mmMNop (97)	Helix C-cap	−65.54, −38.88; −66.34, −29.51
	2	CF	mpCFkl (79)	Coil	−106.09, 133.56; −96.68, 140.72
		CC	mpCCdf (98)	Coil	−106.09, 133.56; −106.09, 133.56
	4	MB	moMBdc (27)	BTVIII	−65.54, −38.88; −92.21, −18.06
	5+	PA	noPAfk (78)	Helix C-cap	59.85, 21.51; −99.80, 131.88
**All-β**	1	BD	dfBDeh (21)	BTIV	−92.21, −18.06; −114.79, 140.11
		PA	koPAcd (52)	BTI, HP3:5, A G	59.85, 21.51; −99.80, 131.88
		KO	dfKOpa (98)	BTI, HP3:5, A G	−59.35, −29.23; −87.27, 5.13
	2	JA	ehJAcc (97)	BTII’, HP2:2	82.88, 150.05; −99.80, 131.88
		JB	ehJBcc (98)	BTII’	82.88, 150.05; −92.21, −18.06
	3	HI	eeHIaf (45)	BTI’,HP2:2	−67.91, 121.55; 77.85, 10.42
		KO	dfKOpa (93)	BTI, HP3:5, A G	−59.35, −29.23; −87.27, 5.13
	4	HI	eeHIaf (66)	BTI’,HP2:2	−67.91, 121.55; 77.85, 10.42
	5+	HI	eeHIaf (57)	BTI’,HP2:2	−67.91, 121.55; 77.85, 10.42
		JA	ehJAcf (59)	BTIV, GTCLA, A C, HP2:2I/2:4	82.88, 150.05; −99.80, 131.88
		KB	dfKBcc (93)	BTI	−59.35, −29.23; −92.21, −18.06
**α/β**	1	PA	noPAcd (47)	Helix C-cap	59.85, 21.51; −99.80, 131.88
		NO	mmNOpa (89)	Helix C-cap	−66.34, −29.51; −87.27, 5.13
		AC	opACdd (89)	Coil	−99.80, 131.88; −106.09, 133.56
	2	PA	noPAcd (55)	Helix C-cap	59.85, 21.51; −99.80, 131.88
		MB	mmMBcc (81)	BT1	−65.54, −38.88; −92.21, −18.06
	3	PA	noPAcd (64)	Helix C-cap	59.85, 21.51; −99.80, 131.88
	4	HI	eeHIac (66)	BTI’,HP2:2	−67.91, 121.55; 77.85, 10.42
		PA	noPAcd (31)	Helix C-cap	59.85, 21.51; −99.80, 131.88
	5+	HI	eeHIac (57)	BTI’,HP2:2	−67.91, 121.55; 77.85, 10.42
		PA	noPAcd (34)	Helix C-cap	59.85, 21.51; −99.80, 131.88
**α+β**	1	OP	mnOPad (30)	Helix C-cap	−87.27, 5.13; 59.85, 21.51
		NO	mmNOpa (86)	Helix C-cap	−66.34, −29.51; −87.27, 5.13
	2	PA	noPAcd (65)	Helix C-cap	59.85, 21.51; −99.80, 131.88
	3	PA	noPAfk (82)	Helix C-cap	59.85, 21.51; −99.80, 131.88
	4	KB	dfKBcc (62)	BT1	−59.35, −29.23; −92.21, −18.06
		HI	eeHIac (67)	BTI’,HP2:2	−67.91, 121.55; 77.85, 10.42
	5+	HI	eeHIac (52)	BTI’,HP2:2	−67.91, 121.55; 77.85, 10.42

The PB bounds (di-PBs) that act as sites for insertions/deletions of different lengths are listed. To obtain a better picture of the local fold, the two PBs that are seen on both sides of the *indel* site were also analysed. The most frequent series are listed and their occurrence frequencies are given in parentheses. PROMOTIF [42] was used for assignment of the local fold corresponding to these frequent PB series. Those regions assigned as coils and are usually found as capping motifs, are labelled as ‘caps’. The following are the local fold definitions implied by the PROMOTIF assignment abbreviations: (see also Table 1). HP*X:Y –* β*-*hairpins, X and Y indicate the number of residues in loop, based on two different rules [42], GTCLA – Classic γ-turns (φ = 75.0±40,ψ = −64.0±40).

The preferences for the site of insertions, has variations across different SCOP classes. A few class specific preferences could be found for the all-α and all-β classes, especially for short inserts of length less than 4 (Table 2). Perhaps, many of the preferred sites for insertions/deletions are class-independent. β-turns and the C-capping region of α-helices are largely found as *indel* sites. These preferred sites are associated with loops that mediate the reversal in the direction of the backbone. Across the different SCOP classes, the two major PB bounds for insertions, are ‘*h-i*’ and ‘*p-a*’. The di-PB ‘*p-a*’ characterizes helix-helix and helix-strand transitions (Figures 8A and D). This local fold is characteristic of the C-cap motif of α-helices. Both short and long insertions are found associated with this site. In the all-β class, this site is preferred for single residue insertions with an association with beta turn of type I (Figure 8B). These di-PB ‘*hi*’ on the other hand, mainly characterizes region of strand-strand transitions (Figures 8B to 8D). Long insertions are found to occur at this site. The local structural region involving ‘*hi*’ is dominated by beta turn of type I’ (Figures 8B to 8D).

Single residue insertions are also preferred in the immediate C-terminus of the regular secondary structural elements. Though short insertions are also frequent in helices (‘*mm*’) and strands (‘*dd*’), the occurrences are not significantly higher than the background.

## Discussion

The precise description of local structures in terms of PBs presents a better view of the preferred local structural differences that occur among homologous proteins. The changes are highly constrained with preferences that are not necessarily correlated with the extent of structural similarity of PBs. β-turns are associated with a significant majority of the conformational variations. This involves both variations within a type of β-turn and exchanges with other types. Conformational flipping between β-turns has been studied for several years, especially inter-conversions between type I and type II turns and between type I’ and II’ [84], [87]. Many of these inter-conversions are noted to be associated with functional interaction and dynamics [88], [89]. Fairly low energy barriers are proposed for these changes and flipping of the central peptide unit (linking C-αs of residues i+1 and i+2) is suggested as a mechanism for these changes [87], [90]. Preferred changes from type I or II to type IV are also seen based on the PB substitution preferences. Replacements between turns and 3._10_ helices also seem to be favoured. In fact, the conformation of 3._10_ helix has similarities with type I β-turn [91]. As the substitution frequencies are calculated from the structurally similar regions, the larger variations are less evident.

Variations in the patterns of local structural changes are observed across different SCOP classes (Figure 5). Specific conformational changes are also preferred in certain SCOP classes (Figure 6). This is most evident in the case of all-β class, where the preferred local structure substitutions are found associated with short helical regions and β-turns. The preferred substitutions involving central helix PB *m* is rather unexpected. Short helices dominate the helical conformations found in the all-β class (Figure S7). About 69.2% of the PB *m* series occurring in this class are of length 3 or lesser. They are often seen in the region of transition between beta strands. Preferred substitutions with the PBs seen in the N-cap of strands (*a* & *c*), usually occur in such regions. Other structural elements associated with preferred local structural differences in the all-β class, are the β-hairpins. This local fold has a very high frequency of occurrence in the all-β class. It is interesting to see that the type IV β-turns are the predominant ones with class specific conformational changes. As they are uncharacterized, they encompass a wide range of conformations.

**Figure 9:**
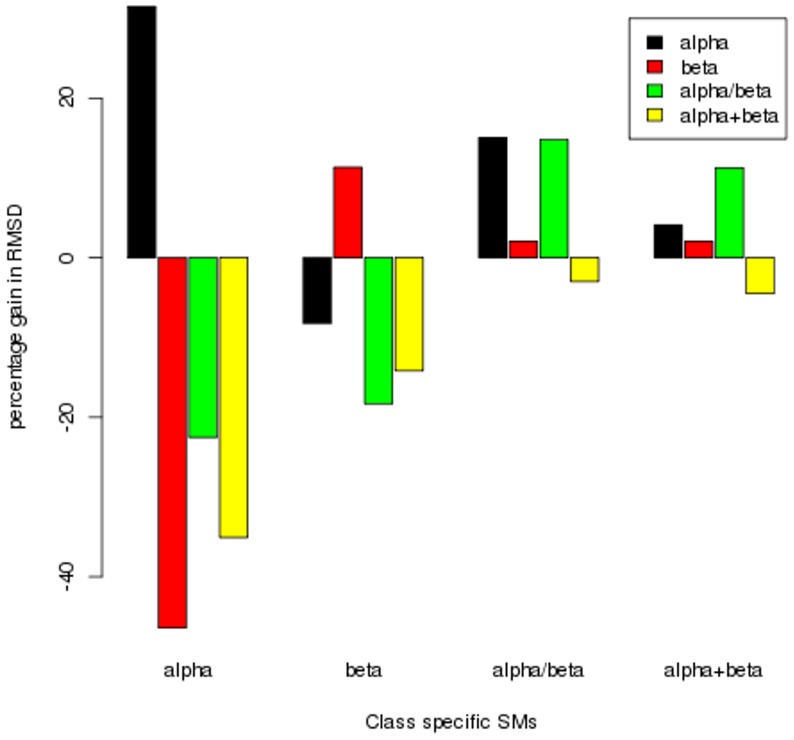
Percentage gain in alignments with better rmsd. Alignment obtained by using class specific PB substitution matrices were compared with that of the global matrix. The percentage of alignments in the dataset with better *rmsd* is plotted. The performance of each class specific SM in each class is highlighted using different colours.

### Using Class Specific PB Substitution Matrices for Structural Alignment

The knowledge on the substitution preferences observed in different SCOP classes could be utilized to improve structural comparisons based on PB sequence alignment [67], [72], [73]. PB based structural alignment method, iPBA, was shown to perform better than other established methods like DALI [92], MUSTANG [81], VAST [93], CE [94] and GANGSTA+ [95]. About 82% of the alignments had better quality when compared to DALI in benchmark tests. Comparable performance could be observed with respect to TMALIGN [96] and FATCAT [97].

The substitution matrices generated from the class-specific datasets are adapted for the background PB composition and observed changes. As seen above, specific domain families were found to contribute a significant portion of PB changes, favoured in a specific class. To avoid this bias resulting from non-uniform distribution of different family sizes, the raw frequencies counted from a family was normalized by the family size. As the substitution matrices are generated using the frequencies from the conserved regions of superposition, it is logical to compare the local alignments obtained using the class specific matrices with respect to the global matrix. The structural alignment pairs in the test dataset were used for this assessment.

As seen on Figure 9, a gain in the alignment quality is achieved in the all-α, all-β and α/β classes, with the use of class specific SMs. With the use of all-α class-specific SM for aligning domains in this class, 50.1% and 30.2% of the structural alignments had better and same rmsd values respectively, when compared to those generated using the general SM. For the all-β class, 38.1% of the alignments were better while 26.8% had poor *rmsd*. For the α/β class 43.3% and 28.8% alignments gave positive and negative results. The α+β class did not show any improvement with the use of specific SM. This suggests that the class specific substitution information could be useful in aligning the structurally similar regions. The negative cases with a lower alignment quality when compared to those generated with the global SM, need to be analysed in detail.

### Hot-spots for Insertions

The relative frequency of occurrence of insertions is similar across different SCOP classes. The distribution of insertion of different lengths in the classes follows similar pattern (Figure S8). However, single residue insertions have a relatively low frequency in the all-β class. The preferred sites of insertions are highly specific in terms of local conformation. Though some class-specific insert sites are observed, the different SCOP classes share many insert sites. Helix C-caps and hairpin turns mainly constitute the sites favourable for occurrence of *indels* (Table 2).

Helix capping motifs have been widely studied since many years and exploring the amino acid preferences associated with these motifs, has been a main area of interest [98], [99], [100], [101], [102]. The dihedral angle distribution of the di-PB ‘*pa*’ is close to that observed in the Schellman motif and the α_L_ type caps [98]. These motifs are stabilized by a specific pattern of backbone hydrogen bonds. Apart from the helix caps, beta turns of types I’, II’ and I are largely seen to characterize the site of *indels.* It is interesting to note that the turns of types I’ and II’ are quite rare, with an occurrence frequency of only about 3% [40]. Hence the preferred insertion sites are largely confined to a few specific conformations.

Both helix caps and beta turns have been implicated in structural stability and protein folding [37], [39], [103], [104], [105], [106], [107]. These β-turn types associated with *indel* sites (Table 2) are characterized by short hairpin loops. The conformation of helix C-caps pertaining to the *indel* sites are also confined to short loops that forms the region of transition with another helix or strand (Figure 8) [98]. These local folds thus restrict the orientation of the flanking secondary structural elements to an antiparallel conformation. The preferred conformation of insert regions is also reported to be shared among turns and coils and most of the *indels* are likely to be tolerated as extensions of the local conformation [30].

The use of dataset specific substitution information has been implicated in the improvement of amino acid sequence alignment [108], [109], [110], [111], [112]. Similar strategy can be adopted in the case of PB based structural alignment too [67], [72], [73]. Class-specific PB substitution matrices have been shown to be useful in improving the quality of alignments pertaining to the class. The nature of specific local structures that act as the hot spots of *indels*, can be also used to develop specialized gap penalties for structural alignment based on PBs. This strategy has already been reported to improve the quality of alignments generated [32], [113].

### Conclusion

Our analysis throws light into the local structure variations that are found among homologous proteins. β-turns are most prone to minor backbone variations and the changes have specificities in certain structural classes. Common differences involve the conformations of types I, II and IV β-turns and to a lesser extent, 3._10_ helices. *Indels* also have preferences for the local structural regions and these preferences vary with the length of the inserted fragment. Short loops involving hairpin β-turns and helix C-caps are the primary targets for insertions. Thus the inserted segments are likely to form structural extensions from these loops. The knowledge on the preferences for conformational variations and *indel* sites also aid in improving the methods for structure comparison and threading. The presence of specific substitution preferences in different structural classes can be explored to improve the PB based structural alignment in the respective class. This work also highlights the use of a structural alphabet which provides an effective description of the local structures of proteins and also gives a different view of the regularities in local conformations.

## Supporting Information

Figure S1 Local structural contexts of (*p,g*) and (*p,i*) substitutions. (A-E) The sites of substitutions involving PBs (*p,g*) and (*p,i*). Some of the frequently occurring penta-PB (5 PB series) changes associated with these substitutions are presented. The change of one penta-PB to another is highlighted using same colours (orange and blue) in the PB series and in the picture.
(DOC)

Figure S2 Some of the frequent local conformational changes associated with the PB *h.* The PB that is structurally closest (angular RMSD) is indicated by black dotted lines. Other PBs that favour substitution with *h* are plotted in different colours.
(DOC)

Figure S3 Comparison of the PB substitution matrix generated from a dataset filtered at 40% sequence identity (A) to the matrices obtained at 60% (B), 80% (C) and also the one without any filtering (D). The substitution scores in each row (associated with each PB) is compared with the respective rows of the other matrix and the correlation coefficients are indicated adjacent to the matrices.
(DOC)

Figure S4 Substitution preferences of PBs classified into buried (*uppercase*) and exposed (*lowercase*). A 32*32 matrix was generated by segregating PBs into buried and exposed, based on a relative solvent accessibility cut-off of 25%. The color scale and corresponding range of substitution scores are given on the right side.
(DOC)

Figure S5 Frequency of occurrence of PBs in various SCOP classes.
(DOC)

Figure S6 The difference in the observed probabilities of substitution in each SCOP class, when compared to the global matrix. Only the observed substitution probabilities were computed for the PB substitutions and their differences from the global probabilities were calculated. This neglects the effect of background frequencies on the substitution scores. For each SCOP class all-α (A), all-β (B), α/β (C) and α+β (D), the variation in the observed probabilities were plotted.
(DOC)

Figure S7 Frequency of occurrence of helical conformation (series of PB *m*) in the all-β class. The percentage of occurrence (y axis) is plotted against the length of PB *m* series (x axis).
(DOC)

Figure S8 Distribution of inserts of different lengths in each SCOP class. The length 5 corresponds to inserts of length greater than or equal to 5.
(DOC)

Table S1 Some of the preferred PB substitutions and the three most frequent secondary structure changes associated with them. The secondary structure assignments were made using DSSP, SEGNO and PROMOTIF (refer Table 1 for details of the assignment abbreviations). The corresponding percentage of occurrence is also given.
(DOC)
