# Supplementary material for: Local Structural Differences in Homologous Proteins: Specificities in Different SCOP Classes
Source: PLoS One. 2012 Jun 22;7(6):e38805. doi: 10.1371/journal.pone.0038805 (PMC3382195; doi:10.1371/journal.pone.0038805)
Supplement: Figure S1 — Local structural contexts of (p,g) and (p,i) substitutions. (A-E) The sites of substitutions involving PBs (p,g) and (p,i). Some of the frequently occurring penta-PB (5 PB series) changes associated with these substitutions are presented. The change of one penta-PB to another is highlighted using same colours (orange and blue) in the PB series and in the picture. (DOC) [file pone.0038805.s001.doc]

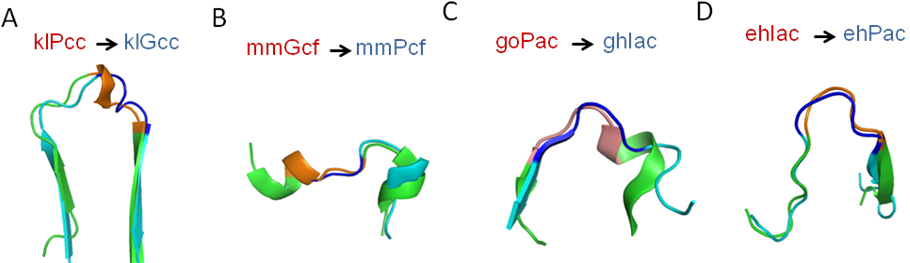


**Figure S1.** Local structural contexts of (*p,g*) and (*p,i*) substitutions. (A-E) The sites of substitutions involving PBs (*p,g*) and (*p,i*). Some of the frequently occurring penta-PB (5 PB series) changes associated with these substitutions, are presented. The change of one penta-PB to another is highlighted using same colours (orange and blue) in the PB series and in the picture.
