# Supplementary material for: Local Structural Differences in Homologous Proteins: Specificities in Different SCOP Classes
Source: PLoS One. 2012 Jun 22;7(6):e38805. doi: 10.1371/journal.pone.0038805 (PMC3382195; doi:10.1371/journal.pone.0038805)
Supplement: Figure S2 — Some of the frequent local conformational changes associated with the PB h. The PB that is structurally closest (angular RMSD) is indicated by black dotted lines. Other PBs that favour substitution with h are plotted in different colours. (DOC) [file pone.0038805.s002.doc]

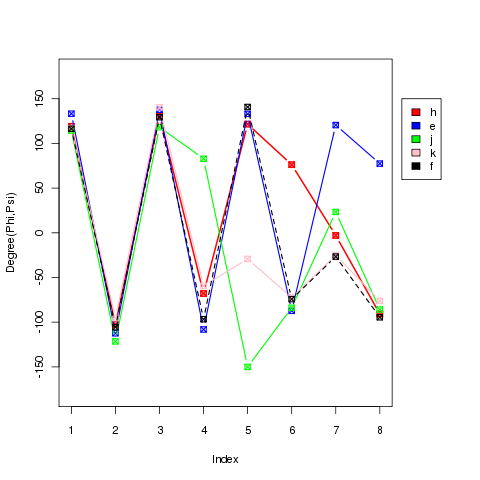


**Figure S2.** Some of the frequent local conformational changes associated with the PB *h.* The PB that is structurally closest (angular RMSD) is indicated by black dotted lines. Other PBs that favour substitution with *h* are plotted in different colours.
