# Supplementary material for: Local Structural Differences in Homologous Proteins: Specificities in Different SCOP Classes
Source: PLoS One. 2012 Jun 22;7(6):e38805. doi: 10.1371/journal.pone.0038805 (PMC3382195; doi:10.1371/journal.pone.0038805)
Supplement: Figure S3 — Comparison of the PB substitution matrix generated from a dataset filtered at 40% sequence identity (A) to the matrices obtained at 60% (B), 80% (C) and also the one without any filtering (D). The substitution scores in each row (associated with each PB) is compared with the respective rows of the other matrix and the correlation coefficients are indicated adjacent to the matrices. (DOC) [file pone.0038805.s003.doc]

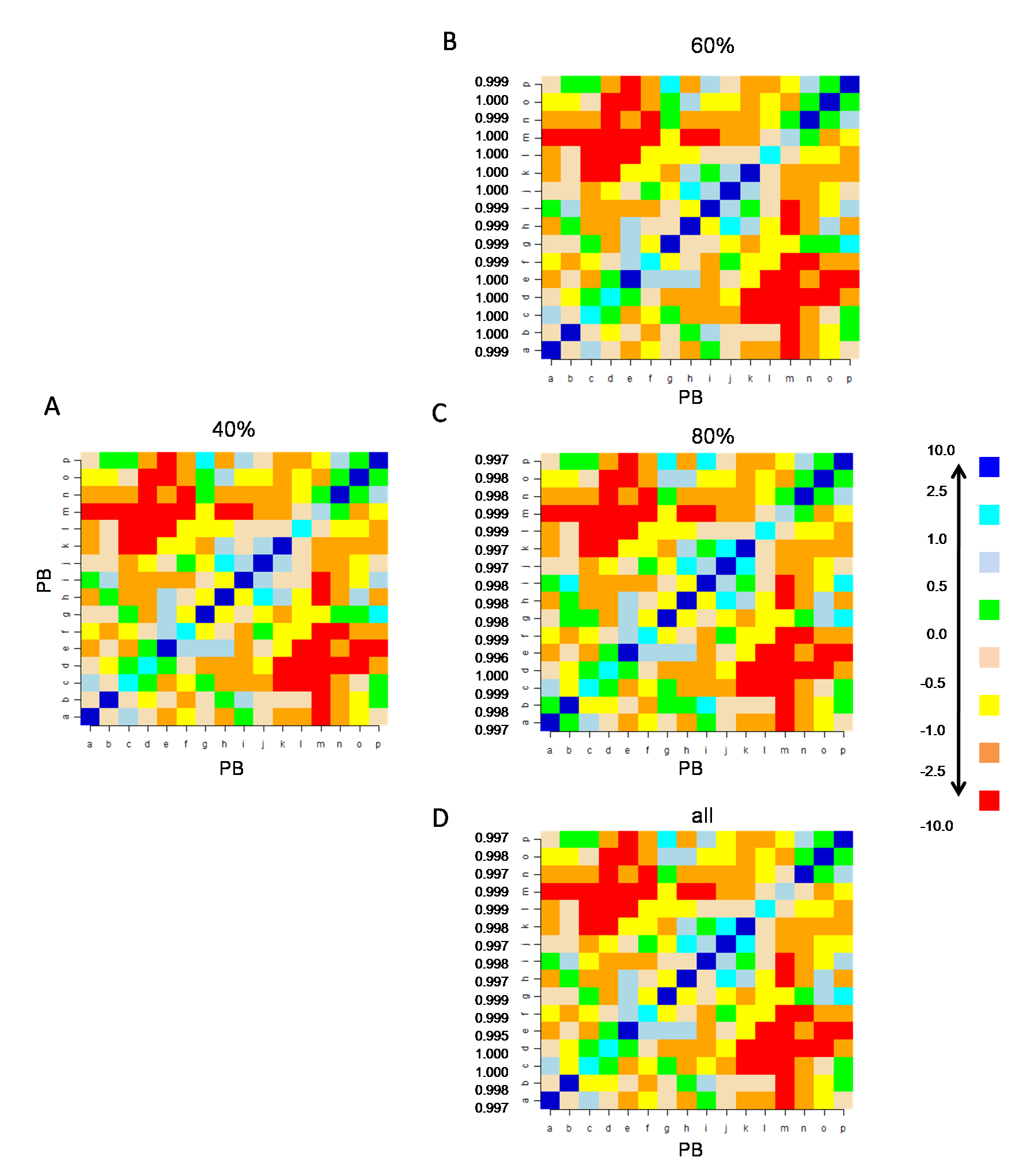


**Figure S3**. Comparison of the PB substitution matrix generated from a dataset filtered at 40% sequence identity (A) to the matrices obtained at 60% (B), 80% (C) and also the one without any filtering (D). The substitution scores in each row (associated with each PB) is compared with the respective rows of the other matrix and the correlation coefficients are indicated adjacent to the matrices.
