# Supplementary material for: Local Structural Differences in Homologous Proteins: Specificities in Different SCOP Classes
Source: PLoS One. 2012 Jun 22;7(6):e38805. doi: 10.1371/journal.pone.0038805 (PMC3382195; doi:10.1371/journal.pone.0038805)
Supplement: Figure S4 — Substitution preferences of PBs classified into buried (uppercase) and exposed (lowercase). A 32*32 matrix was generated by segregating PBs into buried and exposed, based on a relative solvent accessibility cut-off of 25%. The color scale and corresponding range of substitution scores are given on the right side. (DOC) [file pone.0038805.s004.doc]

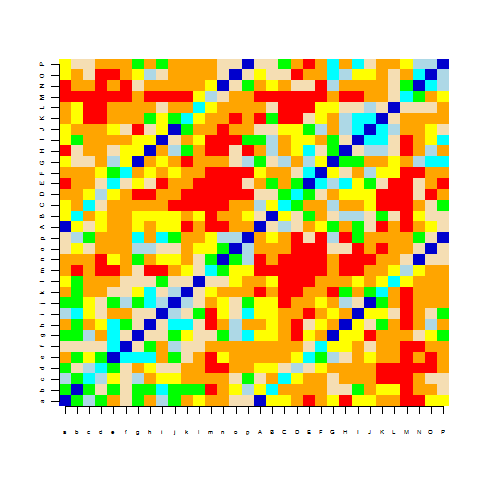


-10.0

-2.5

-1.0

-0.5

0.0

0.5

1.0

2.5

10.0

**Figure S4.** Substitution preferences of PBs classified into buried (*uppercase*) and exposed (*lowercase*). A 32*32 matrix was generated by segregating PBs into buried and exposed, based on a relative solvent accessibility cut-off of 25%. The color scale and corresponding range of substitution scores are given on the right side.
