# Supplementary figures and images for: Local Structural Differences in Homologous Proteins: Specificities in Different SCOP Classes
Source: PLoS One. 2012 Jun 22;7(6):e38805. doi: 10.1371/journal.pone.0038805 (PMC3382195; doi:10.1371/journal.pone.0038805)

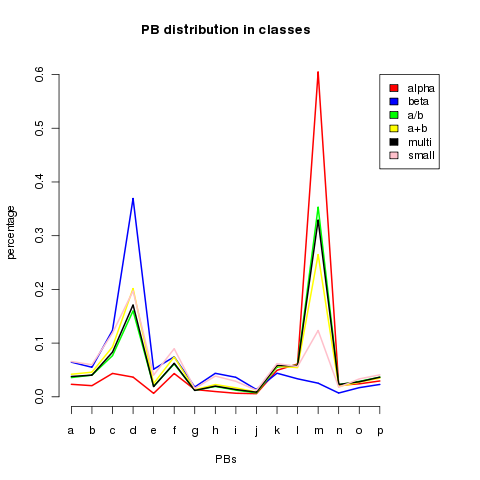


**Figure S5.** Frequency of occurrence of PBs in various SCOP classes

Supplement: Figure S5 — Frequency of occurrence of PBs in various SCOP classes. (DOC) [file pone.0038805.s005.doc]
