# Supplementary material for: Local Structural Differences in Homologous Proteins: Specificities in Different SCOP Classes
Source: PLoS One. 2012 Jun 22;7(6):e38805. doi: 10.1371/journal.pone.0038805 (PMC3382195; doi:10.1371/journal.pone.0038805)
Supplement: Figure S6 — The difference in the observed probabilities of substitution in each SCOP class, when compared to the global matrix. Only the observed substitution probabilities were computed for the PB substitutions and their differences from the global probabilities were calculated. This neglects the effect of background frequencies on the substitution scores. For each SCOP class all-α (A), all-β (B), α/β (C) and α+β (D), the variation in the observed probabilities were plotted. (DOC) [file pone.0038805.s006.doc]

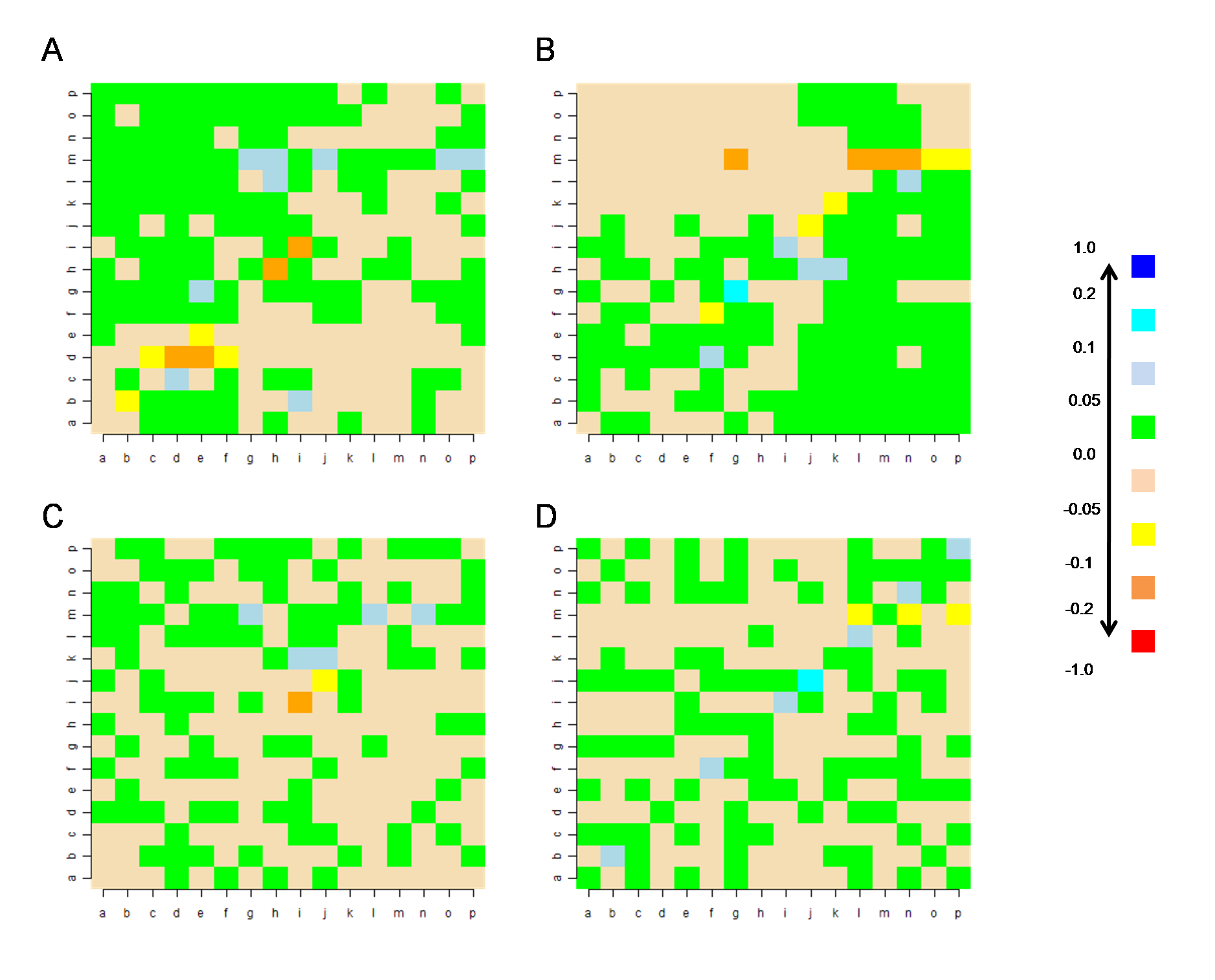


**Figure S6**. The difference in the observed probabilities of substitution in each SCOP class, when compared to the global matrix. Only the observed substitution probabilities were computed for the PB substitutions and their differences from the global probabilities were calculated. This neglects the effect of background frequencies on the substitution scores. For each SCOP class all-α (A), all-β (B), α/β (C) and α+β (D), the variation in the observed probabilities were plotted.
