# Supplementary material for: Local Structural Differences in Homologous Proteins: Specificities in Different SCOP Classes
Source: PLoS One. 2012 Jun 22;7(6):e38805. doi: 10.1371/journal.pone.0038805 (PMC3382195; doi:10.1371/journal.pone.0038805)
Supplement: Figure S7 — Frequency of occurrence of helical conformation (series of PB m) in the all-β class. The percentage of occurrence (y axis) is plotted against the length of PB m series (x axis). (DOC) [file pone.0038805.s007.doc]

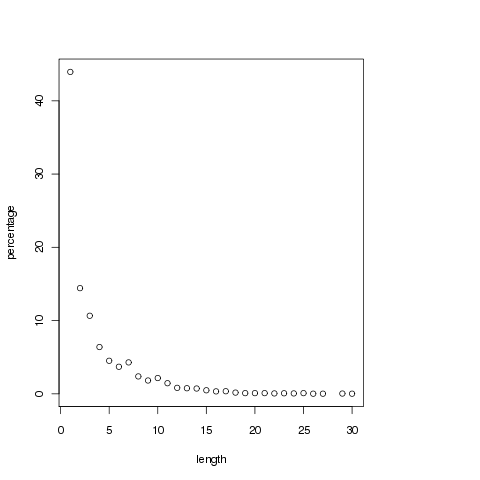


**Figure S7.** Frequency of occurrence of helical conformation (series of PB *m*) in the all-β class. The percentage of occurrence (y axis) is plotted against the length of PB *m* series (x axis).
