# Supplementary material for: Local Structural Differences in Homologous Proteins: Specificities in Different SCOP Classes
Source: PLoS One. 2012 Jun 22;7(6):e38805. doi: 10.1371/journal.pone.0038805 (PMC3382195; doi:10.1371/journal.pone.0038805)
Supplement: Figure S8 — Distribution of inserts of different lengths in each SCOP class. The length 5 corresponds to inserts of length greater than or equal to 5. (DOC) [file pone.0038805.s008.doc]

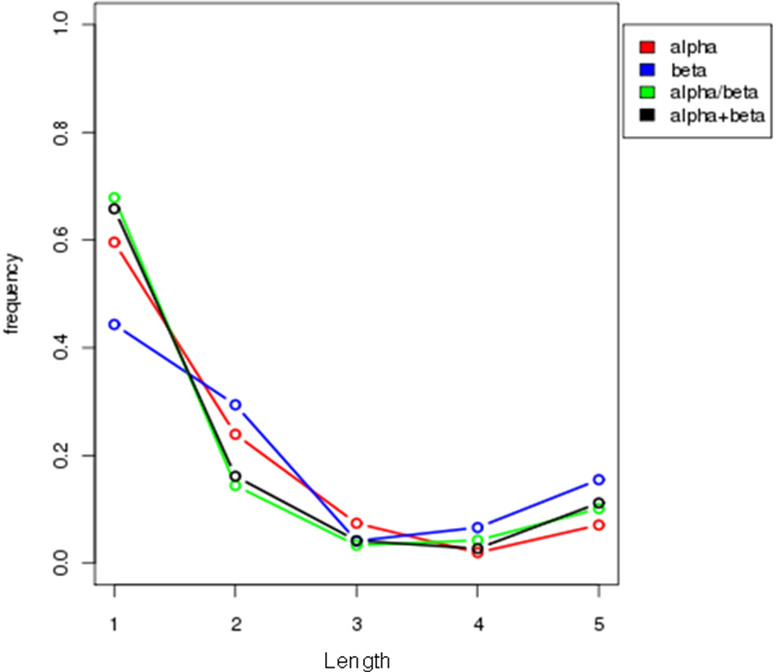


**Figure S8.** Distribution of inserts of different lengths in each SCOP class. The length 5 corresponds to inserts of length greater than or equal to 5.
