# Supplementary material for: Local Structural Differences in Homologous Proteins: Specificities in Different SCOP Classes
Source: PLoS One. 2012 Jun 22;7(6):e38805. doi: 10.1371/journal.pone.0038805 (PMC3382195; doi:10.1371/journal.pone.0038805)
Supplement: Table S1 — Some of the preferred PB substitutions and the three most frequent secondary structure changes associated with them. The secondary structure assignments were made using DSSP, SEGNO and PROMOTIF (refer Table 1 for details of the assignment abbreviations). The corresponding percentage of occurrence is also given. (DOC) [file pone.0038805.s009.doc]

| PB change | DSSP | SEGNO | PROMOTIF |
| --- | --- | --- | --- |
| (*g,p*) | (T,T): 27.8  (G,G): 19.4  (G,T): 6.9 | (C,C): 46.0  (C,G): 13.6  (G,G): 11.8 | (G,G): 19.6  (C,C): 16.2  (BTI,BTI): 8.3 |
| (*i,p*) | (T,T): 30.5  (T,S): 22.2  (S,S): 11.0 | (C,C): 94.3  (C,E): 1.8  (C,G): 1.8 | (BTIV,BTIV): 17.8  (BTII,BTII): 17.7  (BTII,BTIV): 9.0 |
| (*b,i*) | (T,T): 47.5  (S,T): 16.2  (S,S): 11.3 | (C,C): 98.4  (E,C): 0.8  (C,F): 0.5 | (BT1,BTII): 23.1  (BTI,BTIV): 14.2  (BTIV,BTII): 10.7 |
| (*h,j*) | (T,T): 58.4  (S,T): 10.7  (T,S): 10.3 | (C,C): 85.2  (E,C): 5.0  (N,C): 3.8 | (BTIV,BTIV): 48.4  (BTIV,BTII’): 18.1  (BTII,BTIV): 6.1 |
| (*h,k*) | (T,T): 44.0  (T,S): 14.8  (S,T): 6.8 | (C,C): 51.4  (P,C): 14.1  (E,C): 10.6 | (BTII,BTI): 27.0  (BTIV,BTI): 15.6  (BTIV,BTIV): 14.7 |
| (*a,c*) | (E,E): 27.2  (C,C): 27.0  (C,E): 12.2 | (E,E): 37.9  (C,C): 21.8  (C,E): 8.0 | (E,E): 27.4  (BTII,BTI): 7.7  (C,C): 7.3 |

**Table S1.** Some of the preferred PB substitutions and the three most frequent secondary structure changes associated with them. The secondary structure assignments were made using DSSP(1), SEGNO(2) and PROMOTIF(3) (refer Table 1 for details of the assignment abbreviations). The corresponding percentage of occurrence is also given.

1. Kabsch, W. and Sander, C. (1983) Dictionary of protein secondary structure: pattern recognition of hydrogen-bonded and geometrical features. *Biopolymers*, **22**, 2577-2637.

2. Cubellis, M.V., Cailliez, F. and Lovell, S.C. (2005) Secondary structure assignment that accurately reflects physical and evolutionary characteristics. *BMC Bioinformatics*, **6 Suppl 4**, S8.

3. Hutchinson, E.G. and Thornton, J.M. (1996) PROMOTIF--a program to identify and analyze structural motifs in proteins. *Protein Sci*, **5**, 212-220.
